# Supplementary material for: A small RNA that cooperatively senses two stacked metabolites in one pocket for gene control
Source: Nat Commun. 2022 Jan 11;13:199. doi: 10.1038/s41467-021-27790-8 (PMC8752633; doi:10.1038/s41467-021-27790-8)
Supplement: Supplementary file 1 — Supplementary Information [file 41467_2021_27790_MOESM1_ESM.pdf]

Supplementary Information

A small RNA that cooperatively senses stacked metabolites in a single pocket for gene control

Griffin M. Schroeder[1,2], Chapin E. Cavender[1,2], Maya E. Blau[3], Jermaine L. Jenkins[1,2], David H. Mathews[1,2] and Joseph E. Wedekind[1,2]

[1.] *Department of Biochemistry & Biophysics, University of Rochester School of Medicine & Dentistry, Rochester, NY 14642, USA.*

[2.] *Center for RNA Biology, University of Rochester School of Medicine & Dentistry, Rochester, NY 14642, USA.*

[3.] *University of Rochester, 120 Trustee Road, Rochester, NY 14627, USA.*

To whom correspondence should be addressed. Tel: +1 585 273-4516; Email: joseph.wedekind@rochester.edu

Supplementary Table 1: **Data collection and refinement statistics (molecular replacement)**

	$PreQ_1$ Bound *Can* (PDB Entry 7REX)
**Data collection**	
Space group	$P\ 3_2\ 2\ 1$
Cell dimensions	
a = *b*, *c* (Å)	57.8, 153.6
α = β, γ (°)	90, 120
Resolution (Å)	35.8 – 2.60 (2.72 – 2.60)*
R_{merge} (%)	9.6 (88.0)
$R_{P.I.M.}$ (%)	7.1 (66.7)
$I / \sigma(I)$	8.6 (2.0)
Completeness (%)	99.6 (99.1)
Redundancy	4.7 (4.8)
CC1/2	0.99 (0.86)
**Refinement**	
Resolution (Å)	35.8 – 2.60 (2.72 – 2.60)*
No. reflections	17418
R_{work} / R_{free}	0.232/0.272
No. atoms	
RNA	2042
$preQ_1$/ion	78/4
water	1
B-factors (Å^2)	
RNA	87
$preQ_1$	52
r.m.s. deviations from ideal	
Bond lengths (Å)	0.005
Bond angles (°)	0.849
clash score per 1000 atoms	2.5

*Parenthetical values indicate data in the highest resolution shell.

Supplementary Table 2: Average Thermodynamic Parameters for the Wildtype Type I $PreQ_1$-I Riboswitches

Sequence	$K_{D, app}$ (nM)[a]	N	ΔH_1 (kcal mol^{-1})	$-T\Delta S$ (kcal mol^{-1})	ΔG (kcal mol^{-1})
C. antarcticus	32.0 ± 2.0	1.8 ± 0.01	−25.5 ± 0.2	15.3 ± 0.3	−10.3 ± 0.1
H. influenzae[b]	52.9 ± 0.2	2.2 ± 0.03	−25.6 ± 1.1	15.6 ± 1.0	−10.0 ± 0.1
N. gonorrohoeae[b]	50.5 ± 1.3	2.2 ± 0.06	−21.8 ± 1.0	11.8 ± 1.0	−10.0 ± 0.1

[a] *Measured at 25 °C.*
[b] *Classified as type I $preQ_1$-I riboswitch based on Roth et al*[1]

Supplementary Table 3: Average Thermodynamic Parameters for WT and Mutant Type I PreQ$_1$ Riboswitches

Parameter	***Can***[a] **WT**	***Can***[b] **C17U**	***Can***[b] **C31U**	***Hin***[c] **WT**	***Ngo***[d] **WT**
ΔH_{A1} (kcal mol^{-1})	−36.9 (−37.8, −36.3)[e]	−1.6 (−1.8, 0.2)	8.6 (7.6, 9.4)	−40.0 (−40.4, −39.6)	−35.4 (−36.3, −34.6)
ΔH_{B1} (kcal mol^{-1})	−29.9 (−31.4, −28.6)	0.4 (−1.9, 0.8)	−17.7 (−18.7, −15.4)	−26.0 (−26.3, −25.8)	−31.0 (−31.0, −30.9)
ΔH_{A2} (kcal mol^{-1})	−47.4 (−48.8, −45.7)	−4.3 (−4.9, −2.3)	−23.5 (−25.6, −22.4)	−55.1 (−55.5, −54.7)	−37.6 (−39.0, −36.2)
ΔH_{B2} (kcal mol^{-1})	−40.3 (−40.9, −39.5)	−2.3 (−4.4, −2.2)	−49.7 (−51.3, −48.0)	−41.2 (−41.5, −40.9)	−33.2 (−34.7, −31.7)
$K_{D,A1}$ (nM)	1480.0 (1190.0, 1840.0)	5690.0 (5200.0, 6190.0)	9660.0 (8640.0, 11050.0)	3710.0 (3380.0, 4020.0)	1402.0 (1190.0, 1670.0)
$K_{D,B1}$ (nM)	2260.0 (1300.0, 3650.0)	6950.0 (6210.0, 8180.0)	21300.0 (17700.0, 25300.0)	9320.0 (8660.0, 9960.0)	15500.0 (4920.0, 30900.0)
$K_{D,A2}$ (nM)	182.0 (126.0, 273.0)	587.0 (492.0, 803.0)	3200.0 (2430.0, 4180.0)	113.0 (107.0, 120.0)	13.0 (7.0, 39.0)
$K_{D,B2}$ (nM)	278.0 (249.0, 305.0)	716.0 (605.0, 981.0)	7020.0 (6560.0, 7400.0)	284.0 (266.0, 307.0)	142.0 (120.0, 165.0)
C[f]	8.1 (4.5, 14.3)	9.7 (7.3, 11.2)	3.0 (2.6, 3.6)	32.8 (29.7, 35.8)	109.0 (31.0, 249.0)
K_{D1}[g] (nM)	891.0 (630.0, 1208.0)	3130.0 (3030.0, 3220.0)	6640.0 (6420.0, 6830.0)	2650 (2470.0, 2820.0)	1280.0 (974.0, 1580.0)
K_{D2} *(nM)*	461.0 (380.0, 565.0)	1300.0 (1140.0, 1750.0)	10260.0 (9460.0, 10980.0)	398.0 (379.0, 420.0)	156.0 (127.0, 200.0)
γ[h]	7.7 (4.5, 12.6)	9.6 (7.2, 11.0)	2.6 (2.5, 2.7)	26.7 (23.6, 29.6)	32.9 (19.6, 49.1)

[a] *Carnobacterium antarcticus* (*Can*)
[b] ITC was recorded at 25 °C; all other measurements were recorded at 37 °C.
[c] *Haemophilus influenzae* (*Hin*)
[d] *Neisseria gonorrohoeae* (*Ngo*)
[e] Values reported are the median and confidence intervals (2.5, 97.5) from bootstrapping (See **Methods**)
[f] The ratio of microscopic binding constants yields the cooperativity constant C, which shows positive cooperativity when greater than unity.
[g] For simplicity, the microscopic binding constants can be used to generate the macroscopic binding constants, K_{D1} and K_{D2}, corresponding to the first and second ligand binding steps (**Supplemental Fig. 5b**).
[h] The ratio of macroscopic binding constants (K_{D1}, K_{D2}) multiplied by a statistical factor of 4 yields the macroscopic cooperativity constant γ as described in the Methods.

Supplementary Table 4: EC_{50} and fold change in $preQ_1$-induced reporter-gene repression

Riboswitch Sequence	$EC_{50,1}$ (nM)	$EC_{50,2}$ (nM)	EC_{50} Fold Change	Fold Repression
C. antarticus WT[a]	96 ± 14	7100 ± 360	N/A	15.4 ± 1.5
L. rhamnosus WT[b]	15 ± 0.1	N/A	N/A	14.9 ± 1.2
C. antarticus C17U[b]	$4.3 \times 10^5 \pm 1$	N/A	60 ± 3	5.9 ± 0.7
C. antarticus C31U[b]	$1.5 \times 10^6 \pm 1$	N/A	210 ± 10	1.9 ± 0.1

[a] Fit with a biphasic model (see **Methods**).
[b] Fit with log(inhibitor) vs response (three parameters) (see **Methods**).

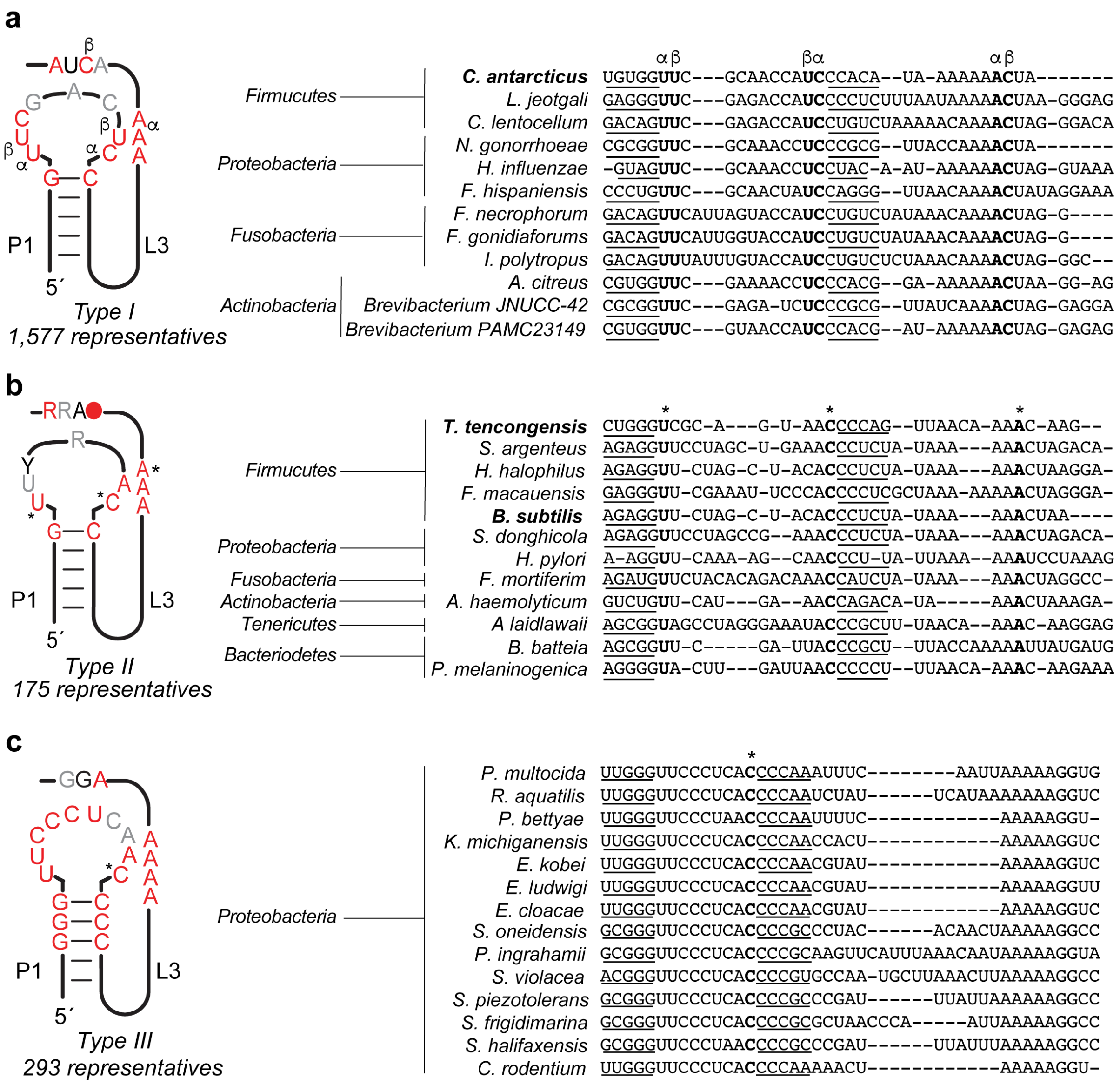

Supplementary Figure 1 | Covariation model and multisequence alignments of preQ$_1$ class I riboswitches. (**a**) Type I Covariation models generated from the full group of known sequence representatives (adapted from[2]); red, black and gray positions indicate 97%, 90% and 75% sequence conservation. Multisequence alignments were generated using a handful of representatives derived from phylogenetically diverse bacteria (reported by McCown et al.[2]). Positions in bold within the alignment recognize preQ$_1$ based on the *C. antarcticus* co-crystal structure of this investigation. PreQ$_1$ binding nucleobases at the α and β sites are each denoted in the covariation model and the sequence alignment as α or β. Here and elsewhere, bolded organisms have been structurally characterized (this work). In addition to the greatest number of representative sequences (indicated in italics), preQ$_1$-I$_I$ riboswitches exhibit the greatest taxonomic diversity[2]. (**b**) same as (**a**), but with type II sequences. Characterized sequences are *T. tencongensis*[3, 4] and *B. subtilis*[5]. Asterisks denote conserved preQ$_1$ recognition positions. (**c**) same as (**a**) and (**b**) but with type III sequences. Due to a lack of structural characterization, the canonical specificity base is the only predicted preQ$_1$ recognition position[2]. Alignments were created in JALVIEW[6].

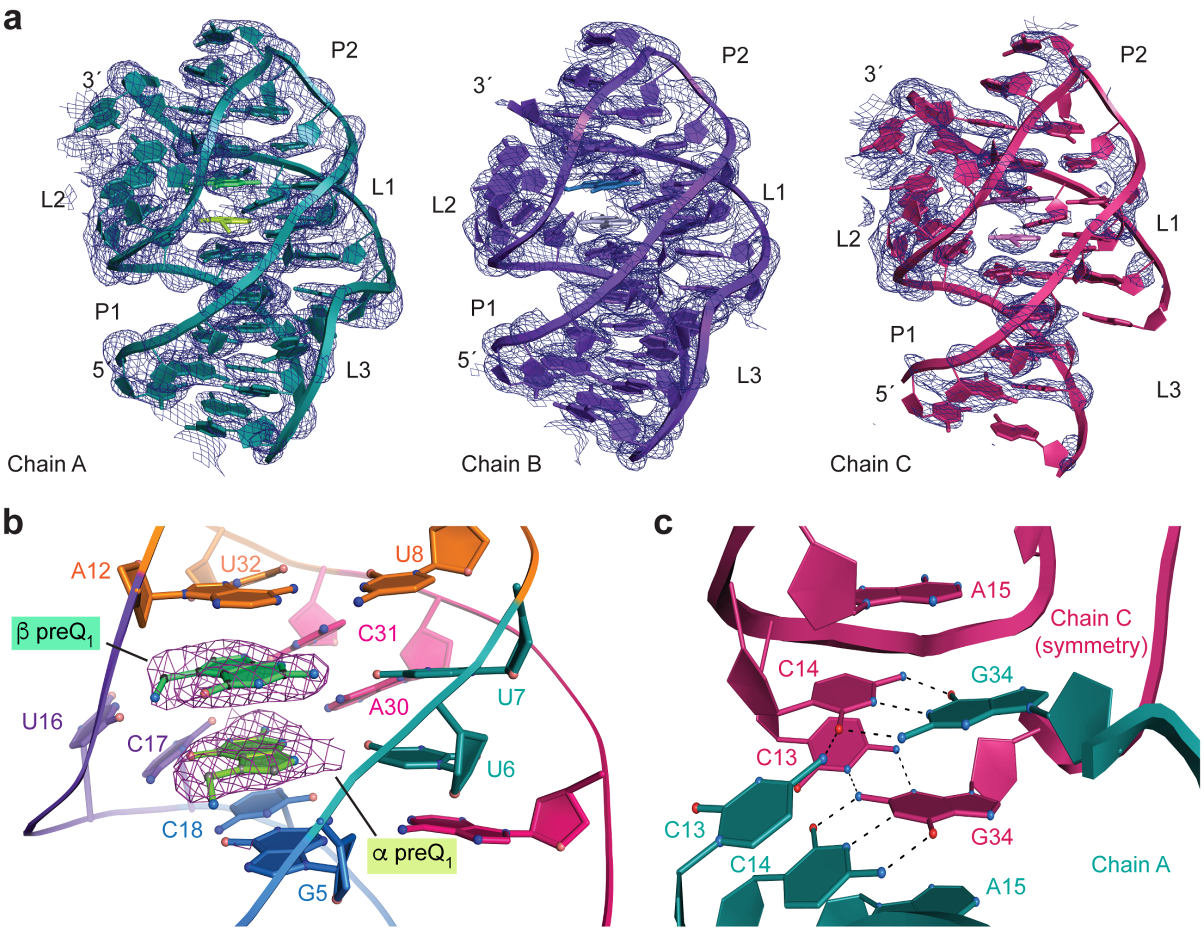

Supplementary Figure 2 | Structural quality and details of the *C. antarcticus* preQ$_1$-I type I riboswitch aptamer and expression platform. (**a**) Reduced bias 2mF_o-DF_c electron-density maps contoured at 1.2 σ around each riboswitch chain in the asymmetric unit. Chains A and B show electron density bathing the entire model, while chain C reveals a break at the junction between P1 and the L3 loop. Individual nucleotides are shown as a cartoon diagram for simplicity. Here and elsewhere, preQ$_1$ is depicted as a ball-and-stick model (green shades in chain A). (**b**) Feature-enhanced, composite-omit map showing the quality of preQ$_1$ models fit to unbiased electron density[7]. (**c**) The expression platforms of chains A and C form a crystal contact that likely takes the place of an intramolecular WC pair between C10 and G34.

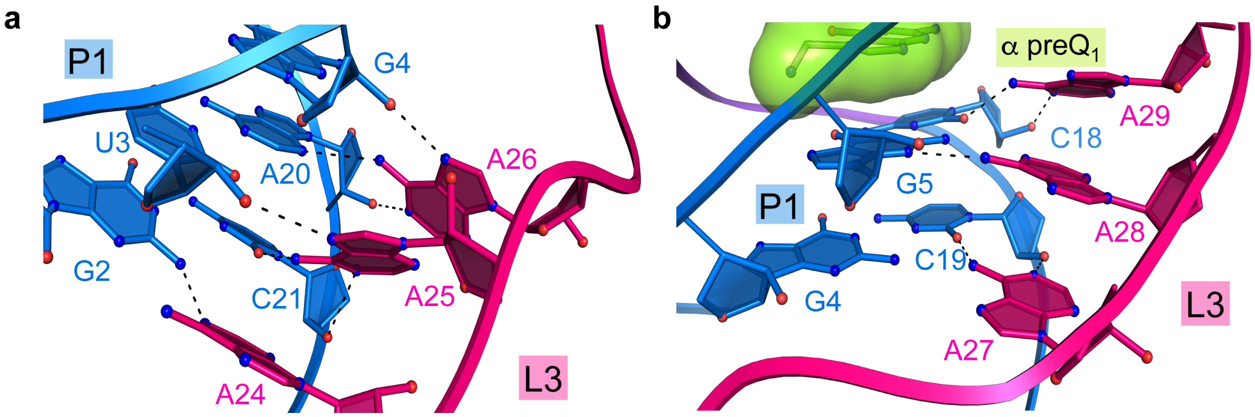

Supplementary Figure 3 | A-amino kissing interactions between Loop 3 and the minor groove of P1. (**a**) View of interactions near the base of P1 where the stem transitions to loop L3. Adenines 24, 25 and 26 engage in sugar edge interactions to P1 via their WC faces and Hoogsteen edges. (**b**) View of the floor of the α-preQ$_1$ binding site. Adenines 27, 28 and 29 use their WC faces to pair with the sugar edges of P1 nucleotides, supporting the floor of the ligand binding pocket.

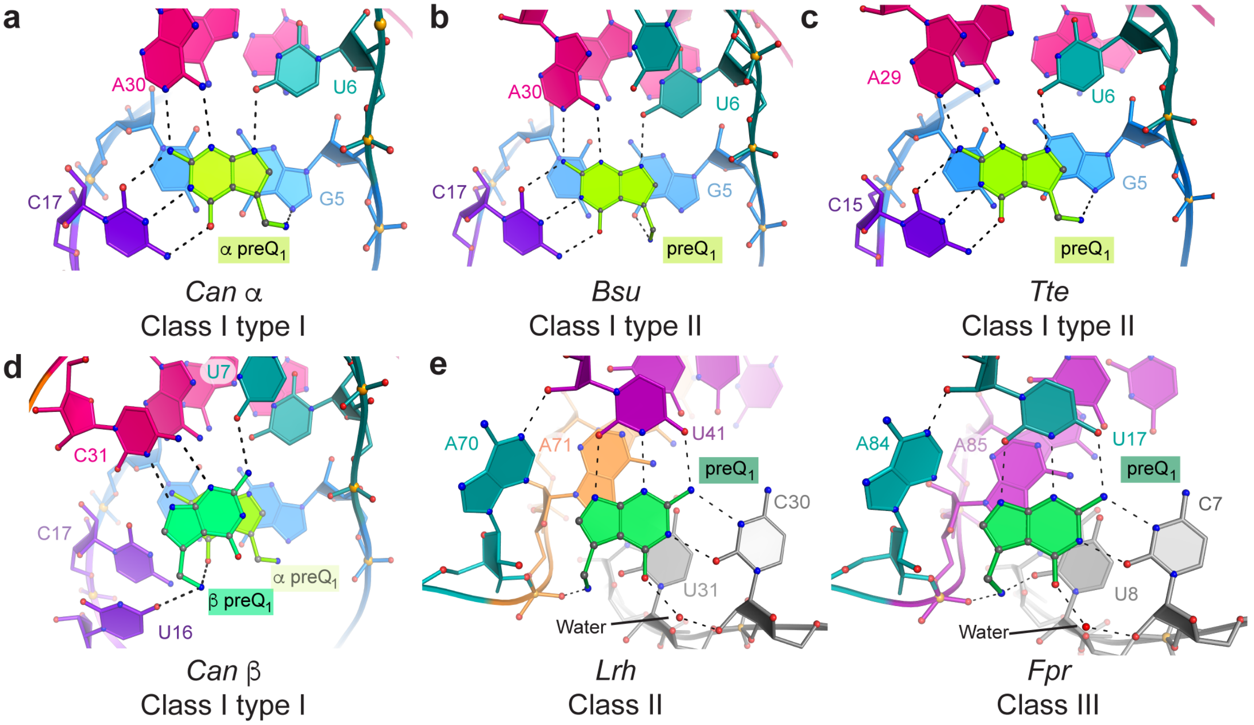

Supplementary Figure 4 | Binding pockets of the *Can* preQ$_1$-I$_I$ riboswitch of this investigation compared to other preQ$_1$ riboswitches. (**a**) The α preQ$_1$ binding site of the *Can* riboswitch. Like known class I structures in (**b**) and (**c**), preQ$_1$ sensing at the α site occurs by canonical *cis* Watson-Crick (WC) pairing. (**b**) Binding pocket of the *B. subtilis* (*Bsu*) class I type II preQ$_1$ riboswitch[5]. Effector readout at this site is equivalent to the *Can* riboswitch α site. (**c**) The binding pocket of the *T. tengcongensis* (*Tte*) class I type II preQ$_1$ riboswitch[4] is equivalent to **b**. (**d**) The β site of the *Can* riboswitch of this investigation displays a new mode of preQ$_1$ recognition of the minor-groove edge equivalent by hydrogen bonds from U7 and C31, whereas the WC face of the ligand is open. (**e**) PreQ$_1$ binding pockets of the class II riboswitch from *L. rhamnosus* (*Lrh*)[8] (*left*) and the class III riboswitch from *F. prausnitzii* (*Fpr*)[9] (*right*). Despite adopting different global folds, both riboswitches use a similar constellation of ten bases for ligand recognition that involves *trans* WC interactions with specificity base C30 or C7. A70 and A84 are inclined A-minor bases that originate from an orthogonal A-form helix that abuts the effector edge[8, 9]. In the preQ$_1$-II riboswitch, these bases are important for gene regulation and dynamics[10-12].

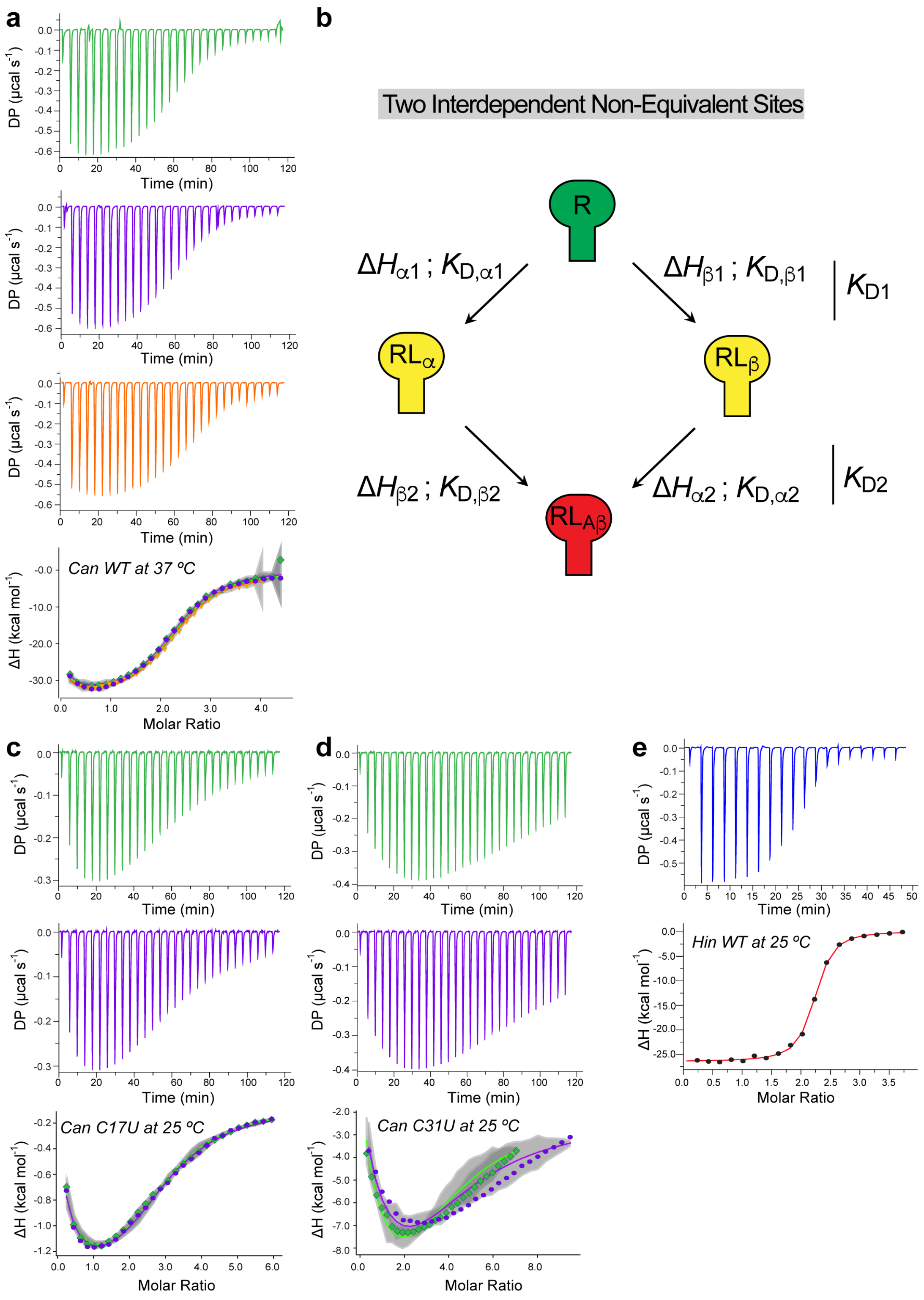
a
b
Two Interdependent Non-Equivalent Sites
R
RLα
RLβ
RLAβ
ΔHα1 ; KD,α1
ΔHβ1 ; KD,β1
KD1
ΔHβ2 ; KD,β2
ΔHα2 ; KD,α2
KD2
Can WT at 37 ºC
c
d
e
Can C17U at 25 ºC
Can C31U at 25 ºC
Hin WT at 25 ºC
DP (µcal s-1)
Time (min)
ΔH (kcal mol-1)
Molar Ratio

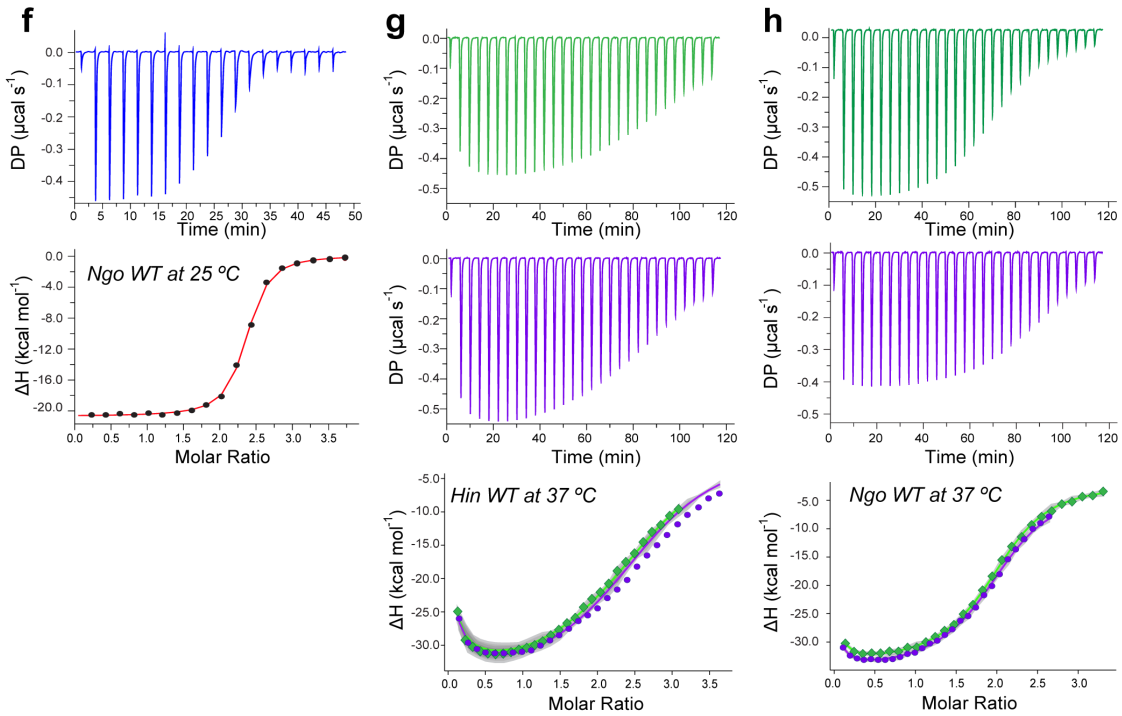

Supplementary Figure 5 | Representative ITC thermograms and corresponding fits: (**a**) Replicate thermograms and global fit of WT *C. antarcticus* (*Can*) riboswitch at 37 °C. Here and elsewhere, the gray areas represent 95% confidence intervals. (**b**) Schematic diagram of the two-interdependent-sites binding model used to fit cooperative isotherms of this investigation (See **Methods**). (**c-d**) Replicate thermograms and global fits of *Can* riboswitch mutants C17U (**c**) and C31U (**d**) at 25 °C. (**e-f**) Representative thermograms of WT *H. influenzae* (*Hin*) (**e**) and *N. gonorrhoeae* (*Ngo*) (**f**) performed at 25 °C and fit with an independent sites (single set of sites) binding model (Malvern Panalytical, Inc). Thermodynamic parameters are listed in **Supplementary Table 2**. (**g-h**) Replicate thermograms and global fits of WT *Hin* (**g**) and *Ngo* (**h**) riboswitches at 37 °C. Cooperative thermograms (**a**, **c**, **d**, **g** & **h**) were analyzed using a non-linear least-squared minimization fitting model developed in our lab (see **Methods**). Thermodynamic parameters for **a**, **c**, **d**, **g** & **h** are in **Supplementary Table 3**. Additional experimental parameters for **e** & **f** are listed in **Supplementary Table 2.**

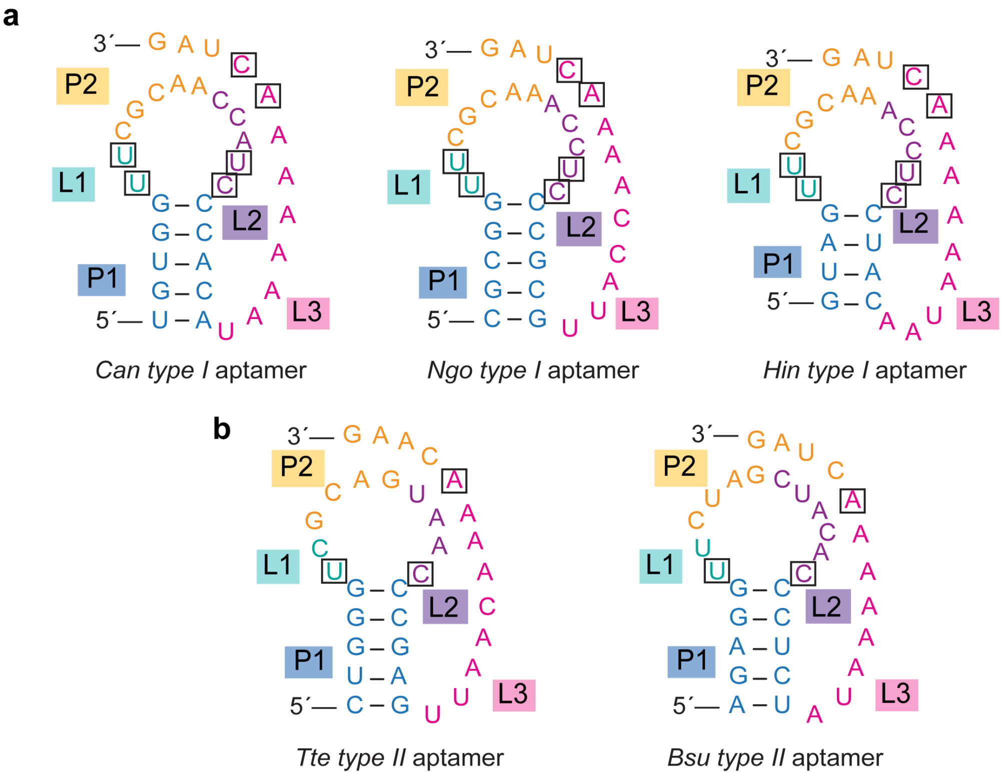

Supplementary Figure 6 | Secondary Structure Models of Known Class I Riboswitches. (**a**) Type I riboswitch sequences from *C. antarticus* (*Can*), *N. gonorrohoeae* (*Ngo*) and *H. influenzae* (*Hin*). The latter two sequences were identified previously[1]. The secondary structure diagram of the *Can* riboswitch was derived from the co-crystal structure of this investigation. Here and elsewhere, positions that contact preQ$_1$ are boxed. Color-codes correspond to specific pseudoknot base pairing (P) and loop (L) sequences as defined[13]. (**b**) Structurally characterized type II aptamers that bind one preQ$_1$ equivalent are from *T. tengcongensis*[3, 4, 14, 15] (*Tte*) and *B. subtilis* (*Bsu*)[1, 5].

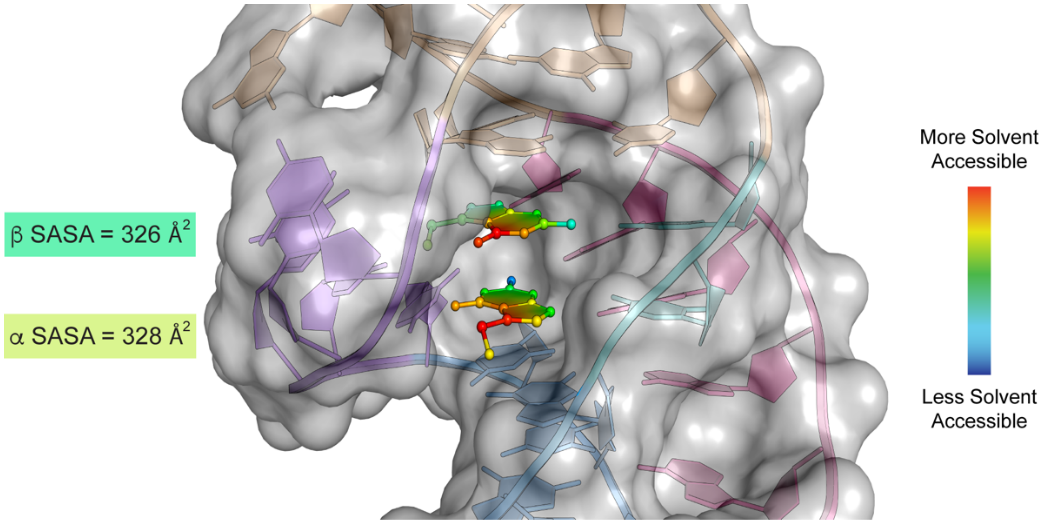

Supplementary Figure 7 | Solvent accessibility of bound effectors. Bound *C. antarcticus* preQ$_1$-I$_I$ riboswitch with the atomic surface shown (semi-transparent gray). The underlying RNA is colored as in **Fig. 1a** with backbone shown as a cartoon for simplicity. Atoms in each preQ$_1$ effector are colored by solvent-accessible surface area (SASA).

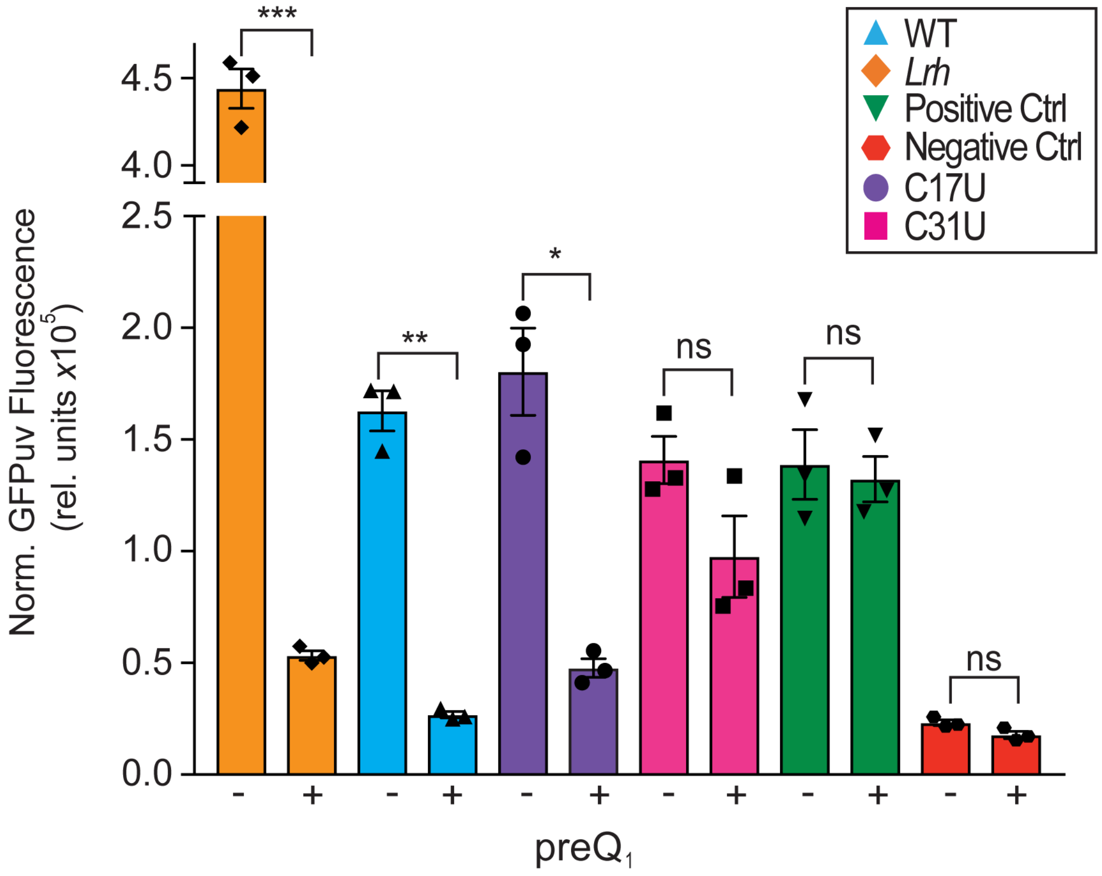

Supplementary Figure 8 | Fluorescence emission for GFP*uv* constructs in live bacteria. Normalized fluorescence emission for each riboswitch construct shows fluorescence emission in the absence of preQ$_1$ for all constructs except the negative control (red). Changes in fluorescence from GFP*uv* in the absence and presence of saturating preQ$_1$ (3 µM) resulted in significantly decreased emission when the reporter gene was under control of WT *L. rhamnosus* (*Lrh*, p = 0.0006) or *C. antarcticus* (*Can*, p = 0.0037) riboswitches; partial reduction of GFPuv emission was observed for *Can* mutants C17U (p = 0.017) and C31U (p > 0.05). The positive and negative controls were unaffected by preQ$_1$. The mean and standard error of the mean (S.E.M.) are reported. Significance was determined by a two-tailed Student's *t*-test with Welch's correction (n = 3 biological replicates. *$p \le 0.05$, **$p \le 0.005$, ***$p \le 0.001$).

Supplementary References

1. Roth, A. et al. A riboswitch selective for the queuosine precursor $preQ_1$ contains an unusually small aptamer domain. *Nat. Struct. Mol. Biol.* **14**, 308-17 (2007).

2. McCown, P. J., Liang, J. J., Weinberg, Z. & Breaker, R. R. Structural, functional, and taxonomic diversity of three $preQ_1$ riboswitch classes. *Chem. Biol.* **21**, 880-889 (2014).

3. Jenkins, J. L., Krucinska, J., McCarty, R. M., Bandarian, V. & Wedekind, J. E., Comparison of a $preQ_1$ riboswitch aptamer in metabolite-bound and free states with implications for gene regulation. *J. Biol. Chem* **286**, 24626-37 (2011).

4. Schroeder, G. M. et al. Analysis of a $preQ_1$-I riboswitch in effector-free and bound states reveals a metabolite-programmed nucleobase-stacking spine that controls gene regulation. *Nucleic Acids Res.* **48**, 8146-8164 (2020).

5. Klein, D. J., Edwards, T. E. & Ferre-D'Amare, A. R., Cocrystal structure of a class I $preQ_1$ riboswitch reveals a pseudoknot recognizing an essential hypermodified nucleobase. *Nat. Struc. Mol. Biol.* **16,** 343-344 (2009).

6. Waterhouse, A. M., Procter, J. B., Martin, D. M. A., Clamp, M. & Barton, G. J., Jalview Version 2—a multiple sequence alignment editor and analysis workbench. *Bioinformatics*, 1189-1191 (2009).

7. Afonine, P. V. et al. FEM: feature-enhanced map. *Acta Crystallogr. D* **71**, 646-666 (2015).

8. Liberman, J. A., Salim, M., Krucinska, J. & Wedekind, J. E. Structure of a class II $preQ_1$ riboswitch reveals ligand recognition by a new fold. *Nat. Chem. Biol.* **9**, 353-355 (2013).

9. Liberman, J. A. et al. Structural analysis of a class III $preQ_1$ riboswitch reveals an aptamer distant from a ribosome-binding site regulated by fast dynamics. *Proc. Natl. Acad. Sci. U. S. A.* **112**, E3485-94 (2015).

10. Dutta, D. & Wedekind, J. E., Nucleobase mutants of a bacterial $preQ_1$-II riboswitch that uncouple metabolite sensing from gene regulation. *J. Biol. Chem.* **295**, 2555-2567 (2020).

11. Soulière, M. F. et al. Tuning a riboswitch response through structural extension of a pseudoknot. *Proc. Natl. Acad. Sci. U. S. A.* **110**, E3256–E3264 (2013).

12. Kang, M., Eichhorn, C. D. & Feigon, J. Structural determinants for ligand capture by a class II $preQ_1$ riboswitch. *Proc. Natl. Acad. Sci. U. S. A.* **111**, E663-E671 (2014).

13. Peselis, A. & Serganov, A. Structure and function of pseudoknots involved in gene expression control. *Wiley Interdiscip. Rev. RNA* **5**, 803-822 (2014).

14. Spitale, R. C., Torelli, A. T., Krucinska, J., Bandarian, V. & Wedekind, J. E. The structural basis for recognition of the $preQ_0$ metabolite by an unusually small riboswitch aptamer domain. *J Biol. Chem.* **284**, 11012-11016 (2009).

15. Kang, M.; Peterson, R. & Feigon, J. Structural Insights into riboswitch control of the biosynthesis of queuosine, a modified nucleotide found in the anticodon of tRNA. *Mol. Cell* **33**, 784-90 (2009).
