# Supplementary material for: A small RNA that cooperatively senses two stacked metabolites in one pocket for gene control
Source: Nat Commun. 2022 Jan 11;13:199. doi: 10.1038/s41467-021-27790-8 (PMC8752633; doi:10.1038/s41467-021-27790-8)
Supplement: Supplementary file 3 — Reporting Summary [file 41467_2021_27790_MOESM3_ESM.pdf]

nature portfolio

Corresponding author(s): Joseph E. Wedekind

Last updated by author(s): Nov 14, 2021

Reporting Summary

Nature Portfolio wishes to improve the reproducibility of the work that we publish. This form provides structure for consistency and transparency in reporting. For further information on Nature Portfolio policies, see our Editorial Policies and the Editorial Policy Checklist.

Statistics

For all statistical analyses, confirm that the following items are present in the figure legend, table legend, main text, or Methods section.

n/a	Confirmed	
☐	☒	The exact sample size (*n*) for each experimental group/condition, given as a discrete number and unit of measurement
☐	☒	A statement on whether measurements were taken from distinct samples or whether the same sample was measured repeatedly
☐	☒	The statistical test(s) used AND whether they are one- or two-sided<br>*Only common tests should be described solely by name; describe more complex techniques in the Methods section.*
☒	☐	A description of all covariates tested
☒	☐	A description of any assumptions or corrections, such as tests of normality and adjustment for multiple comparisons
☐	☒	A full description of the statistical parameters including central tendency (e.g. means) or other basic estimates (e.g. regression coefficient) AND variation (e.g. standard deviation) or associated estimates of uncertainty (e.g. confidence intervals)
☐	☒	For null hypothesis testing, the test statistic (e.g. F, t, r) with confidence intervals, effect sizes, degrees of freedom and P value noted<br>*Give P values as exact values whenever suitable.*
☒	☐	For Bayesian analysis, information on the choice of priors and Markov chain Monte Carlo settings
☒	☐	For hierarchical and complex designs, identification of the appropriate level for tests and full reporting of outcomes
☒	☐	Estimates of effect sizes (e.g. Cohen's d, Pearson's r), indicating how they were calculated

Our web collection on statistics for biologists contains articles on many of the points above.

Software and code

Policy information about availability of computer code

Data collection	Blu-Ice (version 5), Web-Ice (08/2019), PEAQ-ITC Software (version 1.30), MicroCal software for VP-ITC (version 1.04.0024).
Data analysis	XDS (March 15, 2019), CCP4 v7.0.078 (now mentioned and referenced in the manuscript) implementations of Pointless (1.11.21), Aimless (0.7.4), Truncate (7.0.078), Phenix (1.17.1-3660), Coot (0.8.9), PISA (1.52), PEAQ ITC Analysis Software (1.30), Pymol (1.20), In-house ITC Software (deposited into https://github.com/chapincavender/itc_two_site_fit), Prism v7.0e (Graphpad).

For manuscripts utilizing custom algorithms or software that are central to the research but not yet described in published literature, software must be made available to editors and reviewers. We strongly encourage code deposition in a community repository (e.g. GitHub). See the Nature Portfolio guidelines for submitting code & software for further information.

Data

Policy information about availability of data

All manuscripts must include a data availability statement. This statement should provide the following information, where applicable:

- Accession codes, unique identifiers, or web links for publicly available datasets
- A description of any restrictions on data availability
- For clinical datasets or third party data, please ensure that the statement adheres to our policy

Structure factor amplitudes and coordinates for the Can preQ1-II riboswitch were deposited in the Protein Data Bank under accession code 7REX. Publicly available PDB entries used in this study are: 6VUI, 3FU2, 4RZD and 4JF2. Source data files are available in the Supplementary Data, which includes injection data for ITC, and fluorescence emission and cell growth readings for in-cell assays.

March 2021

Field-specific reporting

Please select the one below that is the best fit for your research. If you are not sure, read the appropriate sections before making your selection.

☒ Life sciences ☐ Behavioural & social sciences ☐ Ecological, evolutionary & environmental sciences

For a reference copy of the document with all sections, see nature.com/documents/nr-reporting-summary-flat.pdf

Life sciences study design

All studies must disclose on these points even when the disclosure is negative.

Sample size	Since our approaches do not rely upon sample size considerations, we did not report sample size analysis. The metrics for crystallography used an appropriate level of diffraction data (observations-to-parameters) for refinement. The test set for cross-validation (i.e., Rfree) was based on 10% of the diffraction data chosen at random from the overall data set.
Data exclusions	We did not need to exclude any data sets or data points from the approaches used in the analysis.
Replication	1). ITC measurements were conducted 2 or more times to provide replicate results. All efforts to replicate the results were successful every time. Global fitting analysis using in-house software used multiple titrations shown entirely in Extended Data Figure 4). The concentrations of receptor and ligand used for each ITC experiment are listed completely in the Supplementary Data.<br>2). For bacterial reporter assays, experiments were performed in triplicate using different cell cultures (i.e., biological replicates for each data point). All efforts to replicate the results were successful every time. All data are provided completely in the Supplementary Data.
Randomization	We did not use covariance analysis here, so randomization was not considered. Our approaches do not rely upon making conclusions based upon analysis of groups or populations.
Blinding	Our approach did not require experimental methods that required blinding (i.e., ITC, bacterial reporter assays and X-ray crystallography). Blinding was not necessary because our outcomes were not subject to observer bias. There was no clinical protocol in our approach that might necessitate blinding due to possible bias. Our experiments were designed to measure fundamental binding properties and functional attributes of wildtype and mutant riboswitches. There were no pre-biases since this work has never been conducted before.

Reporting for specific materials, systems and methods

We require information from authors about some types of materials, experimental systems and methods used in many studies. Here, indicate whether each material, system or method listed is relevant to your study. If you are not sure if a list item applies to your research, read the appropriate section before selecting a response.

Materials & experimental systems

n/a	Involved in the study	
☒	☐	Antibodies
☒	☐	Eukaryotic cell lines
☒	☐	Palaeontology and archaeology
☒	☐	Animals and other organisms
☒	☐	Human research participants
☒	☐	Clinical data
☒	☐	Dual use research of concern

Methods

n/a	Involved in the study	
☒	☐	ChIP-seq
☒	☐	Flow cytometry
☒	☐	MRI-based neuroimaging
